# The accuracy of capture per unit effort in predicting density of a cryptic snake was more sensitive to reductions in spatial than temporal coverage

**DOI:** 10.1371/journal.pone.0317764

**Published:** 2025-02-10

**Authors:** Melia G. Nafus, Emma B. Hanslowe, Scott M. Goetz

**Affiliations:** 1 U.S. Geological Survey, Pacific Island Ecosystems Research Center, Hawai‘i National Park, Hawaii, United States of America; 2 National Wildlife Research Center, Wildlife Services, Animal and Plant Health Inspection Services, U.S. Department of Agriculture, Fort Collins, Colorado, United States of America; University of Fribourg, SWITZERLAND

## Abstract

A critical component of monitoring wildlife populations is understanding changes in population size or abundance. However, for most populations a complete census is not possible; thus, trends or abundance need to be estimated through alternative means, such as indexes. An important aspect of using indexes, such as capture per unit effort (CPUE), is validating them as accurate or precise predictors of population trends or abundance. We completed such analyses using data collected from visual surveys and trapping for brown treesnakes (*Boiga irregularis*) within a 5-ha enclosure that was undergoing a continuous population decline. During a ~ 6-year period, we censused and marked the snake population to fully enumerated the population, with new individuals resulting from births and removals resulting only from mortality (natural or experimental). From trapping and visual surveys, we were also able to calculate CPUE as a function of trap nights or km surveyed and used regressions to forecast snake density (snakes/km) in the enclosure from CPUE. We also rarefied the true dataset to measure whether reductions in sampling intensity, either temporally or spatially, affected the accuracy or precision in predicting snake density from CPUE. We found that trap CPUE demonstrated no statistical relationship to density based on our study methods. CPUE during visual surveys did predict actual density, with sufficient spatial and temporal sampling intensity. CPUE from visual surveys was relatively robust against reductions in temporal sampling when spatial intensity remained high. However, reductions in the spatial area covered to less than 50% of the enclosure rapidly reduced the accuracy and precision in using CPUE to forecast density. Our results indicate that visual surveys are a relatively accurate measure of true density for brown treesnakes, given sufficient spatial sampling effort. The spatial area of coverage required for CPUE to accurately predict changes in abundance was, however, intense with > 50% of the spatial area required to be sampled on a given sampling night. Our results indicate that CPUE is only reliable as an index of abundance or population trends for cryptic snakes, if sampling effort covers most of the landscape over which populations are being estimated.

## Introduction

Wildlife management and conservation often requires estimates of trends in species abundance. However, because sampling rarely results in the capture of every individual (*i.e.*, is a complete census), estimates of animal detection or simple counts are often used to model or index population size, respectively [1,2]. For species undergoing monitoring or active management via removals (*e.g.*, invasive species, fisheries, wild game), count- or harvest-based indices can be useful surrogates to censusing or capture-mark-recapture sampling [3–5]. Removal effort is generally represented as a form of capture per unit effort (CPUE), which is a reflection of the number of individuals that were encountered over some defined sampling interval or area searched [6,7]. CPUE is a quantitatively simple index of population trends when captures are verified to correlate with abundance.

The challenges with the application of CPUE to index abundance are well documented through simulations and empirical data [8–11]. Estimates derived from CPUE may be proportional to actual abundance or biased by exhibiting hyperstability or hyperdepletion [11–13]. Hyperstability occurs when CPUE remains high, despite declines in abundance and can be caused by a variety of factors, including the skill of people in capturing individuals [14]. Hyperstability can lead to overestimation of abundance and mask population declines [12,15]. Conversely, hyperdepletion reflects an underestimation in abundance when CPUE declines faster than abundance [13,16]. Optimally, indices of relative abundance would demonstrate a strong linear relationship or correlation with true abundance, as efforts to validate direct relationships can prove them inaccurate [17].

For these reasons, calibration of CPUE metrics against abundance is essential when using them as a metric of population status and trends or efficacy of removal programs [18,19]. For cryptic species with very low individual detection probabilities, alternative indices may be especially important to consider, as populations can be exceptionally difficult to quantify through many typical wildlife monitoring approaches [20,21]. Cryptic reptiles, specifically, have exceptionally low capture probabilities [22], which means the probability of re-encountering individuals during mark-recapture approaches is also low. Capture probabilities are also heterogeneous across individuals and influenced by covariates such as size [23,24]. However, even models that include detection probabilities may not produce useful results [25,26]. These more complex methods, such as mark-recapture, are more limited for cryptic herpetofauna as a result of insufficient recapture rates, whereas other approaches, such as N-mixture models for simple count data, are unreliable [27]. When the goal is to monitor relative population trends, simple metrics such as CPUE, that do not require recapture, may thus have considerable use for indexing abundance, if validated [1,28].

Frequency of sampling may inform overall status trends or index relationships. CPUE can be affected by numerous parameters that extend beyond population status, including environmental conditions, organismal behaviors, or observer variability [14,22,29–32]. Methods implemented need to be designed to help reduce or eliminate temporal, spatial, or observer bias to properly account for these issues. Therefore, a sampling design should sample at sufficient spatial or temporal intervals to capture changes in abundance, while minimizing the potential noise or influence of activity-related metrics.

Brown treesnakes (*Boiga irregularis*) in Guam (known as Guåhan in CHamoru) are an excellent model system for cryptic reptiles, especially snakes. They are a nocturnal, arboreal snake that is a dull brown color that appears vine- or stick-like [33]. Introduced to Guam in the late 1940’s, they caused major vertebrate species declines and extinctions [33–36]. They also have had negative economic and human health effects, including hospitalization of bitten infants and destabilization of the power infrastructure [33,37]. Due to the high risk of their transportation to other islands in the Pacific Region, substantial resources have been invested in controlling brown treesnakes and understanding their detectability or capture probabilities for a variety of control scenarios [38]. Consequently, they are one of the best studied reptiles, particularly snakes, in the world, and much is known about their detection, how individual tools may interface with capture probabilities, and factors that influence them [39–41]. Despite this knowledge and decades of research, there is no statistically viable approach to estimate or forecast population trends for this species. Thus, CPUE is often used as a coarse metric to evaluate the success of management actions.

Given the statistical limitations associated with estimating abundance of cryptic reptiles and the often challenging criterion of having perfect knowledge of true population sizes, direct explorations into relationships between CPUE and abundance in reptiles are difficult to validate. Here, we test relationships between near true abundance and CPUE in brown treesnakes to determine the reliability of the index in forecasting population size based on either trapping or visual survey detection approaches. We also evaluated how survey sampling intensity (temporal and spatial) affected accuracy of indices using data rarefication approaches. We interpret our findings relative to the application of CPUE for indexing cryptic reptile population trends.

## Materials and methods

### Study site

This work was completed in Guam, which is the largest and southernmost island in the Mariana Islands. Guam has a tropical climate, with temperatures ranging from 24–32ºC (Yeo et al. 2023). There are two seasons based on relative rainfall, with peak rainfall occurring from July–October. Cumulative annual rainfall averages 2540 mm [42].

We conducted this work in a 5-ha (224- ×  224-m) enclosure located in Northern Guam (13.639°N, 144.862°E) during 24 October 2016–28 November 2022. The enclosure was constructed of chain-link fence overlaid with mesh to prevent immigration or emigration of snakes into or out of the study site, such that the only causes for changes in population size were birth and mortality. Information on the enclosure is described in detail by Rodda et al. [43], including images of the fence and verification of population “closure.” We trimmed vegetation around the edge of the enclosure on either side of the fence up to approximately 2-m from the fence, including removal of any overhanging branches to prevent alternative escape or access routes over the period of study. Habitat within the enclosure was mixed forest over a limestone karst substrate, and a community structure that was primarily non-native tangantangan (*Leucaena leucocephala*), intermixed with patches of secondary growth limestone forest [44]. We maintained transects in a grid formation throughout the enclosure, such that each transect was approximately 2-m wide, 220-m long, and spaced at roughly 8-m intervals.

### Survey methods

The methods used to census snakes and experimentally remove them are described in depth in Nafus et al. (2022, 2023, 2024). Briefly, we completed visual surveys after sunset, using a powerful headlamp (Wilma, Lupine Lighting Systems, Lebanon, Pennsylvania, USA) by two teams of two observers, per methods described in Christy et al. [22]. Each night of sampling resulted in complete coverage of 29 transects (27 interior forest, 2 edge forest), with each 220-m transect surveyed in 10 min. We completed visual surveys 1-4 times each week, excluding January–March 2019 and March–October 2020 when no surveys occurred. Prior to completing surveys, each observer received 25 hours of training for detecting brown treesnakes in Guam to reduce observer variability. We canceled surveys on nights when rain or wind created unfavorable survey conditions. When a snake was sighted, we stopped time and attempted to capture the individual every time. We resumed surveys after a captured snake was processed.

We also completed censusing using live traps using the protocol described in Terrel et al. [45]. Briefly, a modified minnow trap with a live lure chamber was placed along transects with traps placed in a 16- ×  16-m grid (169 traps). In two instances, March and August 2021, we only trapped along the forest edge [46]. Specific trapping protocols varied in accordance with an effort to first census snakes and then achieve eradication through an adaptive management framework for developing an integrated pest management strategy for this species [46]. Specific trapping periods and intensities are described in Nafus et al. [47]. We processed every trapped snake.

We uniquely marked each newly captured snake using scale-clips and passive integrated transponder tags. We also collected a genetic sample by clipping ~ 1 cm of tail. On subsequent captures, we verified identification codes. On every date of capture and for every captured individual, we collected data on snout-vent length (mm, SVL) and sex. Based on regular captures of individuals, we developed size and sex specific growth rates for this population. We used growth rate estimates to extrapolate presence in the population until birth using the growth rate multiplied against the size at first detection to calculate the first month in which the individual was estimated to be < 400 mm SVL [48]. We selected < 400 mm SVL based on typical documented birth sizes ranging within 300 mm SVL [49]. As an example, a 682 mm female snake first detected in December 2017 was estimated to have a growth rate of 0.98 mm/day and thus was inferred to be present in the population starting in February 2017. Using genetic samples, we also completed parentage analyses on every juvenile to verify that their mother and father were also known individuals [50]. From these data sets, we enumerated the population and had a complete or near complete census during the period of study. The number of snakes present in the population was estimated as the number of snakes known to be present based on a detection on or after a given date and estimated birth month for every individual.

From 24 October 2016–30 March 2017, we censused the population already in the enclosure. On 31 March 2017, we began to remove snakes using an intensive toxic baiting treatment using dead mice laced with 80 mg of acetaminophen [review 46, 48]. From January 2017–February 2021, we released all snakes that we trapped and during March–December 2021, we euthanized snakes captured in traps after collecting morphometric data (Nafus et al. 2024). We released all snakes captured during visual surveys after marking during the period of study described herein. Overall, we conducted a census period of 6 months and then initiated control of snakes interested in rodents or birds from 31 March 2017–28 November 2022.

We completed all animal handling in accordance with Institutional Animal Care and Use Committee protocols through U.S. Geological Survey, Fort Collins Science Center (#2017-03) and Colorado State University (#15‐5892A). No permits were required to complete this work as it was completed under an Interagency Agreement and Memorandum of Understanding between Department of Defense and Department of Interior.

### Statistical Analyses

#### Density and CPUE.

From the total number of snakes known or estimated to be present in the population each month, we calculated the density by dividing the total number of individuals by the enclosure area (snakes per ha). We also calculated the density of snakes susceptible to rodent-based traps by extracting each snake that was > 900 mm SVL in each month and dividing that number by 5 ha. Based on prior data in this study site, all snakes > 900 mm SVL were expected to be susceptible to rodent based traps [43]. We calculated CPUE for visual surveys as a function of cumulative snakes captured per km of visual surveys and trapping as the cumulative snakes captured per corrected trap night. We averaged CPUE across the total distance of transects or trap nights sampled throughout a given 1-month period to reduce searcher or trap bias effects and minimize the effect of temporal autocorrelation. Data described in this manuscript therefore represent monthly estimates of population size and averaged CPUE data, not daily estimates. Raw data and processed data to support month population size estimates and CPUE are publicly accessible [47,51]. Based on needing multiple months to detect presence of individuals, we did not model data past 27 September 2022 and used October and November 2022 data primarily as a validation of which snakes remained present in the population through September 2022.

We used a Poisson regression to test whether average monthly CPUE predicted snake density (*N* =  40 sampling points) using visual survey or trapping CPUE. Because of the likelihood of a non-linear relationship, we tested a linear and a quadratic term for CPUE, and predicted density from CPUE with 95% confidence intervals using the best fit model. We used a chi-square goodness of fit test to verify a Poisson distribution. As a baseline, we estimated that 29 transects, which represented 1–2-m of cut vegetation, was equivalent to spatial coverage of approximately 51% of the 5-ha enclosure assuming a 1-m detection area on either side of the transects. We did not measure detection distance, because distance sampling is inaccurate for brown treesnakes, but rather assumed that snakes within 1 m of the transect were as reliably detectable as a brown treesnake is by human observers [52,53].

#### Temporal Sampling Configuration (Visual Surveys).

We rarefied the full dataset to reduce sampling depth to estimate how a reduction in temporal frequency of sampling affected the relationship between visual survey CPUE and known density. For all temporal models we kept spatial sampling set at 51% of the enclosure. We then selected three baseline sampling intervals: monthly (M), quarterly (Q), and bi-annually (B). Monthly sampling required a survey to be completed every month of the year. Quarterly sampling required surveys only approximately every 3 months. Bi-annual sampling was defined as two discrete survey periods occurring roughly 6 months apart. Within each M, Q, and B surveys, we also selected five distinct sampling intensities and structures: two visual surveys per week for four consecutive weeks (M1, Q1, B1); one visual survey per week for four weeks (M2, Q2, B2); one visual survey per week for two consecutive weeks (M3, Q3, B3); two visual surveys in a single week, once in a 30-day period (M4, Q4, B4); and one visual survey in a 30-day period (M5, Q5, B5), yielding 15 sampling schemes (Table 1). To rarefy the data, we randomly selected a start date in October 2016 and then automated the removal of surplus data to fit the structure of each of the 15 sampling schemes. For example, if 4 nights of visual surveys were completed for 8 weeks, then a start day in the first week was randomly selected and the remaining 3 nights deleted and so on for each month in the M2 model. We filtered periods without adequate survey effort relative to specified criteria from the analyzed data (*i.e.*, a week without two visual surveys during that week was excluded as a selection for a M4, Q4, or B4 model). For M models, we used a negative binomial model and included a quadratic term to predict abundance based on CPUE. For Q and B models, we used a linear term for CPUE because there were fewer sampling points, which limited the number of factors. We used a chi-square goodness of fit test to verify model distribution. We calculated the average residual distance and standard deviation from predicted and actual density as a measure of precision and accuracy in model forecasts.

**Table 1:** Visual survey schemes based on sampling interval. The number of days (total survey days) for which visual surveys met the criteria for five sampling schemes that we simulated as monthly (every month: M), quarterly (1 month out of 90 days: Q), and biannually (1 month out of 180 days: B) based on model order (number). Weeks sampled per unit frequency (M, Q, or B) was the number of weeks surveyed within a given month and days surveyed was the number of days surveyed within a given week. All surveys were for brown treesnakes in a 5-ha enclosure in northern Guam during 24 October 2016–27 September 2022.

Model Number	Weeks survey per month	Days surveyed per week	Total survey days
Monthly (M): Each month			
M1 ^*^	4	2	167
M2	4	1	106
M3	2	1	109
M4	1	2	303
M5	1	1	72
Quarterly (Q): Once per 3 months			
Q1 ^*^	4	2	113
Q2	4	1	65
Q3	2	1	51
Q4	1	2	49
Q5	1	1	27
Biannual (B): once per 6 months			
B1	4	2	67
B2	4	1	41
B3	2	1	26
B4	1	2	28
B5	1	1	14

* We selected models for use in temporal rarefication simulation.

#### Spatial Sampling Configuration (Visual Surveys).

For two of the top temporal sampling configurations (*i.e.*, those that generated predicted populations that most accurately tracked the known population) at the monthly and quarterly level, we artificially reduced the spatial coverage to 25% (14 transects), 9% (5 transects), 4% (2 transects), and 1% (1 transect) of the enclosure. In all instances, we used a repeated sampling design such that we resampled selected data from the same transects across time. We filtered transects non-randomly to maintain sampling evenly spread in the field site (i.e., if the transects were labeled as ‘A, B, C, D… CC’ for 29 transects, a reduction to 14 was evenly distributed to exclude data from alternating transects, such that we sampled transects ‘A, C, E…’). We used negative binomial regressions to predict the density of snakes from the relationship between true density and CPUE under each sampling framework. We used a chi-square goodness of fit test to verify model distribution. We calculated the average residual distance and standard deviation from predicted and actual density as a measure of precision and accuracy of model forecasts.

## Results

### CPUE as a Predictor of Density

**Fig 1:**
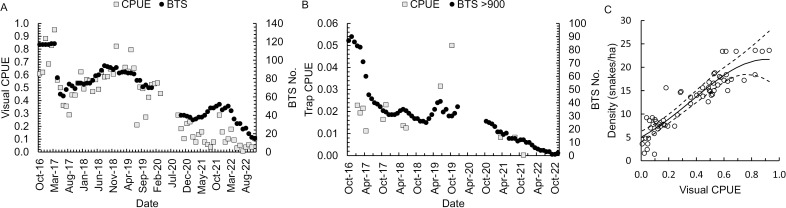
The number of brown treesnakes (BTS) and capture per unit effort (CPUE) in a 5-ha enclosure. BTS numbers are the total number of snakes in the enclosure relative to CPUE from nocturnal visual surveys (snakes/km, A), the number of BTS > 900 mm snout-vent length (SVL) and trap CPUE (snakes/trap night, B), and the Poisson regression curve and 95% confidence interval, depicting the predicted relationship between visual survey CPUE and snake density (total snakes/ha, C). Open circles represent actual data points.

Overall, we completed 523 nights of visual surveys and surveyed a cumulative distance of 6,250 km from October 2016–November 2022. We also completed 10 trapping bouts from January 2017–23 December 2021, resulting in a cumulative of 27,374 trap nights. Total snake density ranged from 2.4–23.4 snakes/ha (15–117 individuals), and visual survey CPUE ranged from 0.008–0.928 snakes/km (Fig 1A). During the trapping intervals, snake density for individuals > 900 mm SVL varied from 2–17 snakes/ha (10–85 individuals) and trap CPUE varied from 0.0003–0.05 snakes/night (Fig 1B). We found no relationship between trap CPUE and density of snakes > 900 mm SVL (Table 2). CPUE during visual surveys was, however, predictive of snake density, with a quadratic polynomial function (Table 2), such that each increase in CPUE of approximately 0.01 correlated with an increase in density by a factor of 1 (Fig 1C).

**Table 2:** Poisson and quadratic Poisson regression models for monthly averages of brown treesnake density (snakes/ha) as predicted by capture per unit effort (CPUE). CPUE data are for snake captures during visual surveys (snakes/km) or trapping (snakes/trap night), and density (snakes/ha) is for either all snakes (visual) or only snakes > 900 mm snout-vent-length (SVL, trap) in a 5-ha enclosure in Northern Guam during October 2016–September 2022. β is the model parameters, with β_0_ equal to the model intercept, ‘BTS.CPUE’ is the additive change from brown treesnake CPUE, with the upper (U) and lower (L) 95% confidence interval (CI) around the β for CPUE from snake trapping (trap) or visual surveys (visual). The data from visual surveys included a quadratic term based on a non-linear relationship between CPUE and snake density. Z is the z-score for each model factor.

	β	95% CI		z
		L	U	
Trap				
β_0_	0.35	-0.62	1.32	0.74
BTS.CPUE	0.02	-0.05	0.10	0.46
Visual				
β_0_	2.45	2.39	2.52	62.3
BTS.CPUE	3.58	3.03	4.13	11.4
BTS.CPUE^2^	-0.82	-1.15	-0.49	-3.14

### Temporal Sampling Configuration (Visual CPUE)

**Fig 2:**
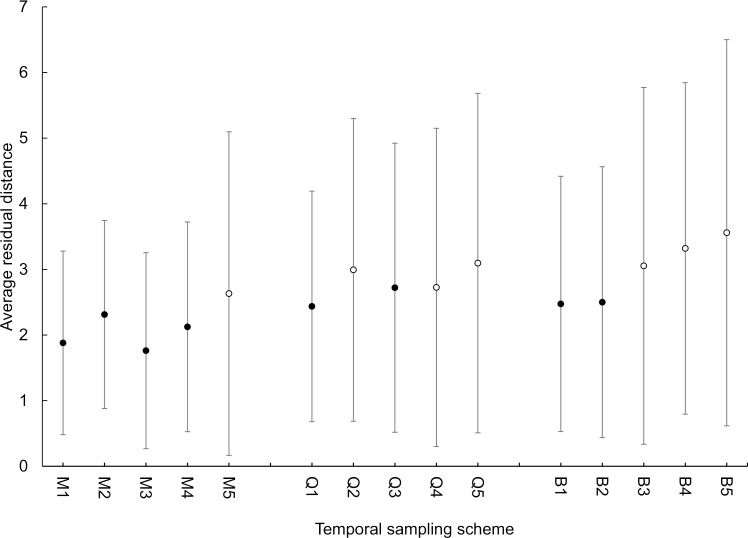
The average residual distance between the predicted and actual densities ( ** ±** **1 standard deviation) of brown treesnakes (*Boiga irregularis*) per ha.** Snake density was predicted across 15 temporal visual survey sampling configurations covering > 50% of a 5-ha enclosure in Northern Guam during October 2016–September 2022. M1-M5 represent monthly sampling, Q1-Q5 are quarterly sampling, and B1-B5 are biannual sampling. Within each frequency weekly and nightly surveys ranged from 2 nights per week for 4 weeks (1), 1 night per week for 4 weeks (2), 2 nights surveyed bi-weekly (3), 2 nights surveyed within 1 week (4), and 1 night per month (5). Black circles represent models that met the criteria of having in an average residual distance + 1 standard deviation (SD) totaling < 5 and open circles were models where average residual distance + 1 SD was > 5.

We tested 15 sampling designs varying across 14–303 days of surveys spread over the monitoring period (Table 1). Overall, all 15 models were reasonably accurate in predicting the density of snakes based on CPUE, with the average residual distance from true density tending to be < 3 snake/ha (Fig 2). However, the precision across models varied greatly with some models having a > 10 snakes/ha in the difference between predicted and actual densities in each month, and standard deviation in residual distance ranging up to 6 snakes/ha (Fig 2). Across all three sampling intervals, the models with the greatest precision where those that sampled two nights per week each week for four continuous weeks (M1, Q1, B1), based on the average residual distance between actual and predicted density across sampling occasions (Fig 2).

**Fig 3:**
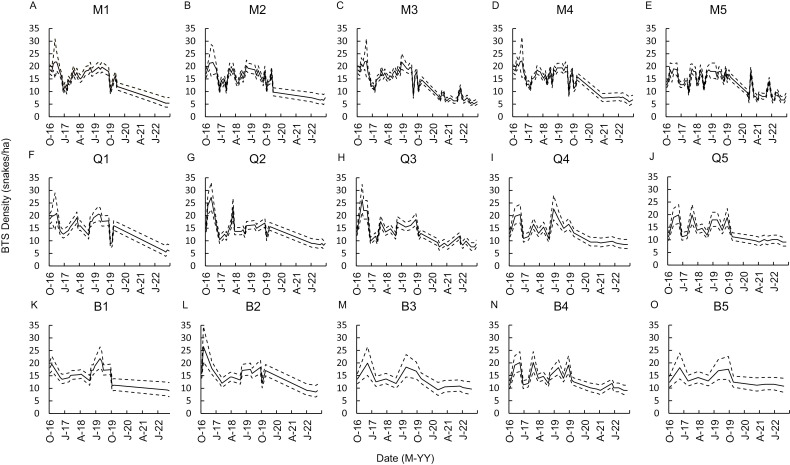
The predicted density of brown treesnakes based on capture estimates from different temporal sampling intensities. Predicted (solid line, dashed lines are 95% confidence interval) abundance of brown treesnakes (*Boiga irregularis*) based on capture per unit effort during visual surveys covering > 50% of a 5-ha enclosure, using Poisson regression on a declining population monitored from October 2016–September 2022. Effort included monthly (A-E), quarterly (F-J), and biannual (K-O) surveys at 2 nights per week for 4 weeks (A, F, K), 1 night per week for 4 weeks (B, G, L), 1 night per week bi-weekly (C, H, M), 2 nights surveyed within 1 week (D, I, N), or 1 night per month (E, J, O).

For models with a monthly sampling structure, sampling more than once per month appeared to be important for predicting population trends across time using CPUE (Fig 3). Sampling periods as few as 2 survey nights within a 2-week period yielded density predictions similar to actual density and appeared predictive of true density, as well as population trends (Fig 3A and 3B). However, by the time sampling was reduced to a single night each month, the predicted and actual values no longer aligned sufficiently to detect population changes (Fig 3E). Similar trends were reflected by quarterly sampling approaches, whereas biannual sampling yielded comparatively unreliable estimates of population density and trends (Fig. 3K–3O).

### Spatial Sampling Configuration (Visual CPUE)

**Fig. 4:**
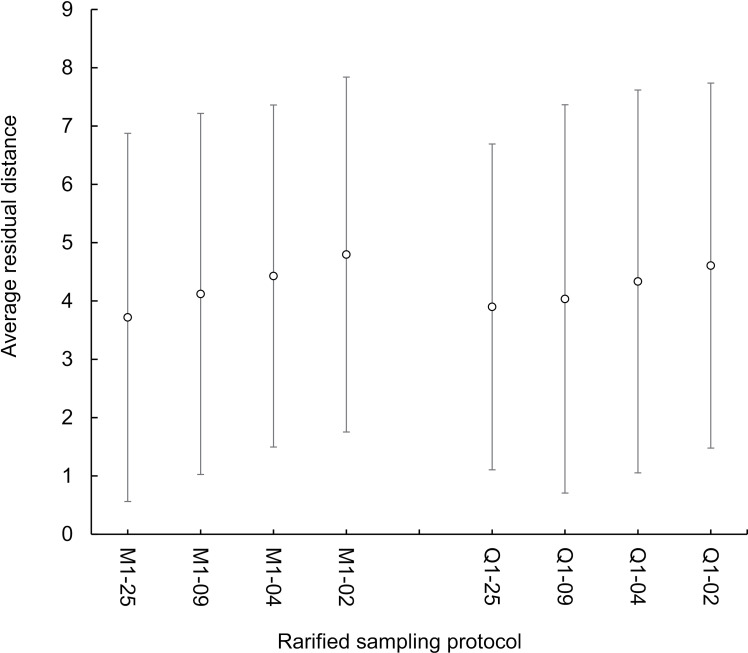
The average residual distance between the predicted and actual densities ( ** ±** **1 standard deviation) of brown treesnakes (*Boiga irregularis*)/ha predicted by 4 spatial structures for visual survey sampling configurations.** Sampling was completed in a 5-ha enclosure on Northern Guam during October 2016–September 2022 at monthly (M1) or quarterly (Q1) sampling intervals, with surveys occurring 2 nights each week per month sampled. Spatial area covered included 25% (25), 9% (09), 4% (04), and 2% (02) of the 5-ha enclosure. Open circles indicate that models all reflected an average residual distance + 1 standard deviation that was > 5 snakes from the true density.

**Fig. 5:**
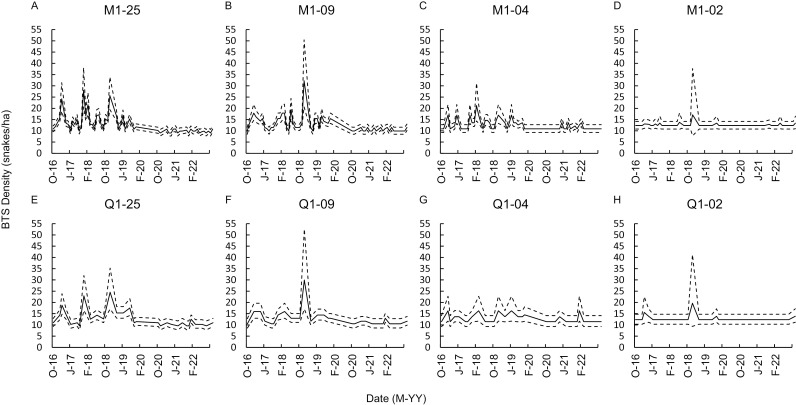
The predicted density of brown treesnakes (*Boiga irregularis*) based on spatial sampling configuration. Predicted (solid black line, dashed lines are 95% confidence interval) density of brown treesnakes based on capture per unit effort during visual surveys using negative binomial analysis of a declining population in a 5-ha enclosure monitored from October 2016–September 2022. We rarefied data to model a reduction in transects surveyed to represent 25% of the enclosure (A, E, -25), 9% of the enclosure sampled (B, F, -09), 4% of the enclosure sampled (C, G, -04), or only 2% of the area surveyed (D, H, -02). We modeled surveys as monthly (M1: A, B, C, D) or quarterly (Q1: E, F, G, H) schedules, with 2 survey nights for 4 weeks per month sampled.

We selected M1 and Q1 temporal models, as judged by accuracy and precision of residual deviance, to use for evaluating the effects of reduced spatial sampling. Accuracy of density predictions was highly sensitive to reductions in the spatial scale of sampling, with large distances between predicted and actual density of snakes (Fig 4). Reductions down to < 4% of the habitat sampled yielded CPUE based predictions that were unsuccessful in tracking either the true density or the population trends across time at the monthly and quarterly sampling schedules (Fig 5). CPUE predicted density estimates predicted that the population was stable when the snakes were declining.

## Discussion

Our study was informative in that we had near perfect knowledge of abundance based on intensive mark-recapture surveys during an intentional effort to reduce the population. We were therefore able to directly compare actual density to predicted density from CPUE to determine the relative levels of accuracy or precision in predicting abundance or population trends based on survey approach. Based on these data, CPUE from visual surveys can be a reasonable technique to estimate density or monitor population trends for brown treesnakes in Guam with sufficient effort. With regards to visual surveys, a quadratic term was supported, demonstrating a curvilinear relationship [54]. In contrast, trapping CPUE demonstrated no statistical relationship to density, indicating that either our sampling lacked sufficient intensity or factors other than abundance or density are driving trap captures of snakes. Although prior studies have found that every brown treesnake > 900 mm SVL can be captured in a trap with sufficient effort [43], the reliability of this relationship may be somewhat dependent on a lack of prey available on the landscape [55,56]. Visual surveys thus appeared more reliable than traps in estimating trends in brown treesnake density.

On account of stringent visual survey protocols that, in essence, attempted to control for detection probability, the positive quadratic relationship between brown treesnake CPUE and density may carry over to similar localities in Guam and represent relative trends in snake density over space and time [28]. Specifically, we observed that sampling configurations over a varying degree of temporal schematics remained relatively precise and accurate in predicting density. However, a reduction in the spatial extent of the area covered from 50% to even 25% in essence negated the ability to use CPUE to predict density or monitor population trends. Of course, the method by which we classified area covered is prone to introducing bias in its own way. For example, we limited the calculation of estimated spatial area covered during surveys to within 1 m of the 2-m transect, but snakes can be detected farther than 1 m from a transect [52]. Thus, the spatial coverage necessary to monitor population trends based on count data at this site was extensive, at > 50% of the area surveyed each night. This scale of sampling would be difficult to achieve in most study applications and reinforces the idea that occupancy-based surveys may be a more reliable approach to monitor trends in species that are difficult to detect [27].

Maximizing the spatial area covered was more critical for maintaining the relationship between CPUE and density than surveying over a greater number of days. The accuracy of count-based abundance or density indices relies on the assumption that detection probability is constant across observers, space, time, and individual or groups of animals [18,57,58]. Consequently, indices are criticized when they are applied to and compared among scenarios where detection may fluctuate or differ from seminal validation studies or when unvalidated. CPUE for brown treesnakes may vary as a result of spatial variation in prey availability, which may affect movement or activity levels of individual snakes [16,59,60]. Therefore, application of our models to index brown treesnake density beyond our study site, especially in non-forest habitats, ideally would account for or parameterize effects of habitat or food on detection estimates.

In this study, errors between predicted and actual densities indicated hyperstability, and snake density was overpredicted when populations were declining or at low density. In fisheries and wild game management, where the goal is long-term population viability in conjunction with harvest, hyperstability masks declines in abundance and can lead to population crashes [12]. Alternatively, hyperstability may safeguard invasive species control if inflated indices prolong search and/or removal efforts to ensure density is low enough or, optimally, zero. However, this is not without the potential tradeoff of additional costs associated with potentially unnecessary efforts, if the population is indeed already extirpated and required search times to verify this occurrence are then overestimated. Despite the ongoing debates regarding the utility of count-based abundance or density indices [28,57,58], they remain widely used across taxa and systems because they are relatively simple to implement and interpret. Many harvest-based indices rely on data reported by members of the public (*i.e.*, hunters and fishers) and are useful when appropriately corrected or validated [61]. Additionally, CPUE is applied in citizen-science based data and can be more informative for population estimation than non-effort corrected or presence-only data [62].

In general, understanding the ideal spatial or temporal scale to accurately monitor population trends is essential for the application of index-based techniques for wildlife management. Including spatial-temporal modelling approaches may be an important part of accurately using CPUE to predict population changes, as wildlife are not evenly distributed across the landscape [63]. Additionally, CPUE remains a cost-effective strategy to assess population trends in many species, especially on small islands (Jessop et al. 2007). However, many factors can affect capture probabilities including weather and body size, and those may in turn affect variables such as CPUE [22,30,31,64]. Understanding error as a product of sample design is critical for the application of tools such as CPUE or capture data in monitoring populations. For instance, our results indicate that with finite resources, prioritizing visual surveys over a larger spatial area for a shorter period of time may yield more accurate trends than completing more surveys in less area over more nights. However, our data indicate that with insufficient spatial extent coverage, CPUE is not a reliable index for brown treesnake density or population trends and that how individuals are captured is also important for monitoring population trends.

## Conclusions

Overall, extremely intensive sampling allowed for a direct and predictive effect of CPUE on absolute density from visual surveys but not trapping. CPUE for snakes from visual surveys was affected more by reductions in spatial coverage than reduction in the number of surveys across time. The spatial area covered to generate accurate predictions of density was likely more intensive than would be practical or feasible for most monitoring areas regarding visual surveys. Thus, CPUE is not a reliable metric of population density or trends unless the spatial scale of sampling is intensive.

## References

[1] Skalski JR, Ryding KE, Millspaugh JJ. Analysis of population indices. In: Skalski JR, Ryding KE, Millspaugh JJ, editors. Wildlife Demography. Burlington: Academic Press; 2005. p. 359-–433.

[2] Rodda GH. Population size and demographics. In: McDiarmid RM, Foster M, Guyer C, Gibbons JW, Chernoff N, editors. Reptile biodiversity: Standard methods for inventory and monitoring. Berkeley, CA: University of California Press; 2012. p. 283-–322.

[3] Cattadori IM, Haydon DT, Thirgood SJ, Hudson PJ. Are indirect measures of abundance a useful index of population density? The case of red grouse harvesting. Oikos. 2003;100(3):439–46. doi: 10.1034/j.1600-0706.2003.12072.x

[4] Roseberry JL, Woolf A. A comparative evaluation of techniques for analyzing white-tailed deer harvest data. Wildl Monogr. 1991;117(117):3–59.

[5] Ueno M, Solberg EJ, Iijima H, Rolandsen CM, Gangsei LE. Performance of hunting statistics as spatiotemporal density indices of moose (Alces alces) in Norway. Ecosphere. 2014;5(2):1–20. doi: 10.1890/es13-00083.1

[6] Ormerod SJ, Tyler SJ, Pester SJ, Cross AV. Censussing distribution and population of birds along upland rivers using measured ringing effort: a preliminary study. Ring Migr. 1988;9(2):71–82. doi: 10.1080/03078698.1988.9673928

[7] Jessop TS, Madsen T, Ciofi C, Jeri Imansyah M, Purwandana D, Rudiharto H, et al. Island differences in population size structure and catch per unit effort and their conservation implications for Komodo dragons. Biol Conserv. 2007;135(2):247–55. doi: 10.1016/j.biocon.2006.10.025

[8] Crecco V, Overholtz WJ. Causes of density-dependent catchability for Georges bank haddock Melanogrammus aeglefinus. Can J Fish Aquat Sci. 1990;47(2):385–94. doi: 10.1139/f90-040

[9] Rose GA, Leggett WC. Effects of biomass–range interactions on catchability of migratory demersal fish by mobile fisheries: an example of Atlantic cod (Gadus morhua). Can J Fish Aquat Sci. 1991;48(5):843–8. doi: 10.1139/f91-100

[10] Gillis DM, Peterman RM. Implications of interference among fishing vessels and the ideal free distribution to the interpretation of CPUE. Can J Fish Aquat Sci. 1998;55(1):37–46. doi: 10.1139/f97-206

[11] Harley SJ, Myers RA, Dunn A. Is catch-per-unit-effort proportional to abundance?. Can J Fish Aquat Sci. 2001;58(9):1760–72. doi: 10.1139/f01-112

[12] Erisman BE, Allen LG, Claisse JT, Pondella DJ II, Miller EF, Murray JH. The illusion of plenty: hyperstability masks collapses in two recreational fisheries that target fish spawning aggregations. Can J Fish Aquat Sci. 2011;68(10):1705–16. doi: 10.1139/f2011-090

[13] Roa-Ureta RH. Modelling in-season pulses of recruitment and hyperstability-hyperdepletion in the Loligo gahi fishery around the Falkland Islands with generalized depletion models. ICES J Mar Sci. 2012;69(8):1403–15. doi: 10.1093/icesjms/fss110

[14] Ward HGM, Askey PJ, Post JR. A mechanistic understanding of hyperstability in catch per unit effort and density-dependent catchability in a multistock recreational fishery. Can J Fish Aquat Sci. 2013;70(10):1542–50. doi: 10.1139/cjfas-2013-0264

[15] Rose GA, Kulka DW. Hyperaggregation of fish and fisheries: how catch-per-unit-effort increased as the northern cod (Gadus morhua) declined. Can J Fish Aquat Sci. 1999;56(S1):118–27. doi: 10.1139/f99-207

[16] Alós J, Campos-Candela A, Arlinghaus R. A modelling approach to evaluate the impact of fish spatial behavioural types on fisheries stock assessment. ICES J Mar Sci. 2018;76(2):489–500. doi: 10.1093/icesjms/fsy172

[17] Rodda GH, Dean-Bradley K, Campbell EW, Fritts TH, Lardner B, Yackel Adams AA. Stability of detectability over 17 years at a single site and other lizard detection comparisons from Guam. J Herpetol. 2015;49(4):513–21.

[18] Nichols JD. Capture-recapture models. BioScience. 1992;42(2):94–102. doi: 10.2307/1311650

[19] Guzzo MM, Rennie MD, Blanchfield PJ. Evaluating the relationship between mean catch per unit effort and abundance for littoral cyprinids in small boreal shield lakes. Fish Res. 2014;150:100–8. doi: 10.1016/j.fishres.2013.10.019

[20] Durso AM, Willson JD, Winne CT. Needles in haystacks: estimating detection probability and occupancy of rare and cryptic snakes. Biol Conserv. 2011;144(5):1508–15. doi: 10.1016/j.biocon.2011.01.020

[21] Dorcas ME, Willson JD. Innovative methods for studies of snake ecology and conservation. In: Stephen JM, Richard AS, editors. Snakes: Ecology and conservation. Ithaca, NY: Cornell University Press; 2011. p. 5-–37.

[22] Christy MT, Yackel Adams AA, Rodda GH, Savidge JA, Tyrrell CL. Modelling detection probabilities to evaluate management and control tools for an invasive species. J Appl Ecol. 2010;47(1):106–13. doi: 10.1111/j.1365-2664.2009.01753.x

[23] Koons D, Birkhead R, Boback S, Williams M, Greene M. The effect of body size on cottonmouth (Agkistrodon piscivorus) survival, recapture probability, and behavior in an Alabama swamp. Herpetol Conserv Biol. 2009;4 221–35.

[24] Nicolau PG, Sørbye SH, Yoccoz NG. Incorporating capture heterogeneity in the estimation of autoregressive coefficients of animal population dynamics using capture-recapture data. Ecol Evol. 2020;10(23):12710–26. doi: 10.1002/ece3.6642 33304489 PMC7713978

[25] Steen DA. Snakes in the grass: secretive natural histories defy both conventional and progressive statistics. Herpetol Conserv Biol. 2010;5:183–8.

[26] Joseph LN, Field SA, Wilcox C, Possingham HP. Presence-absence versus abundance data for monitoring threatened species. Conserv Biol. 2006;20(6):1679–87. doi: 10.1111/j.1523-1739.2006.00529.x 17181803

[27] Ward RJ, Griffiths RA, Wilkinson JW, Cornish N. Optimising monitoring efforts for secretive snakes: a comparison of occupancy and N-mixture models for assessment of population status. Sci Rep. 2017;7(1):18074. doi: 10.1038/s41598-017-18343-5 29273793 PMC5741746

[28] Engeman R. More on the need to get the basics right: population indices. Wildl Soc Bull. 2003;31:286–7. doi: 10.2307/3784386

[29] Hoyle S, Langley A, Campbell R. Recommended approaches for standardizing CPUE data from pelagic fisheries. WCPFC Scientific Committee. 2014.

[30] Araujo MA, Romaire RP. Effects of water quality, weather and lunar phase on crawfish catch1. J World Aquac Soc. 1989;20(4):199–207. doi: 10.1111/j.1749-7345.1989.tb01003.x

[31] Lardner B, Rodda GH, Adams AAY, Savidge JA, Reed RN. Detection rates of geckos in visual surveys: turning confounding variables into useful knowledge. J Herpetol. 2015;49(4):522–32. doi: 10.1670/14-048

[32] Hauser CE, Giljohann KM, McCarthy MA, Garrard GE, Robinson AP, Williams NSG, et al. A field experiment characterizing variable detection rates during plant surveys. Conserv Biol. 2022;36(3):e13888. doi: 10.1111/cobi.13888 35098569 PMC9303269

[33] Rodda GH, Savidge JA. Biology and impacts of Pacific Island invasive species: Boiga irregularis, the brown tree snake (Reptilia: Colubridae). Pac Sci. 2007;61(3):307–24.

[34] Savidge JA. Extinction of an island forest avifauna by an introduced snake. Ecology. 1987;68(3):660–8. doi: 10.2307/1938471

[35] Wiles GJ, Bart J, Beck RE JR, Aguon CF. Impacts of the brown tree snake: patterns of decline and species persistence in Guam’s avifauna. Conserv Biol. 2003;17(5):1350–60. doi: 10.1046/j.1523-1739.2003.01526.x

[36] Rodda GH, Fritts TH. The impact of the introduction of the brown tree snake, Boiga irregularis, on Guam’s lizards. J Herpetol. 1992;26 166–74.

[37] Fritts TH. Economic costs of electrical system instability and power outages caused by snakes on the Island of Guam. Int Biodeterior Biodegradation. 2002;49(2–3):93–100. doi: 10.1016/s0964-8305(01)00108-1

[38] Engeman RM, Vice DS. Integr Pest Manage Rev. 2001;6(1):59–76. doi: 10.1023/a:1020441405093

[39] Rodda GH, Fritts TH. Sampling techniques for an arboreal snake, *Boiga irregularis*. Micronesica. 1992;25(1):23–40.

[40] Rodda G, Fritts T, Clark C, Gotte S, Chiszar D. A state-of-the-art trap for the brown treesnake. Problem Snake Management: The Habu and Brown Treesnake. 1999 268–305.

[41] Rodda GH, Dean-Bradley K. Size selectivity of brown treesnake traps. Micronesica. 2004;37:180–1.

[42] Yeo M-H, Patil UD, Chang A, King R. Changing trends in temperatures and rainfalls in the western pacific: Guam. Climate. 2023;11(4):81. doi: 10.3390/cli11040081

[43] Rodda GH, Savidge JA, Tyrrell CL, Christy MT, Ellingson AR. Size bias in visual searches and trapping of brown treesnakes on Guam. J Wildl Manag. 2007;71(2):656–61. doi: 10.2193/2005-742

[44] Nafus MG, Savidge JA, Yackel Adams AA, Christy MT, Reed RN. Passive restoration following ungulate removal in a highly disturbed tropical wet forest devoid of native seed dispersers. Restor Ecol. 2017;26(2):331–7. doi: 10.1111/rec.12559

[45] Tyrrell CL, Christy MT, Rodda GH, Yackel Adams AA, Ellingson AR, Savidge JA, et al. Evaluation of trap capture in a geographically closed population of brown treesnakes on Guam. J Appl Ecol. 2009;46(1):128–35. doi: 10.1111/j.1365-2664.2008.01591.x

[46] Nafus MG, Reyes A, Fies T, Goetz SM. Adaptive resource management (ARM): achieving functional eradication of invasive snakes to benefit avian conservation. J Appl Ecol. 2024;61:733–45.

[47] Nafus MG, Collins AF, Viernes MC, Hopkins CB, Nacpil A. Guam, USGS Closed Population (NWFN), an experimental eradication of brown treesnakes in a 5-ha study site, 2016 - 2023. ScienceBase. 2023.

[48] Nafus MG, Siers SR, Levine BA, Quiogue ZC, Yackel Adams AA. Demographic response of brown treesnakes to extended population suppression. J Wildl Manag. 2022;86:1–19.

[49] Mathies T, Miller LA. Cool temperatures elicit reproduction in a biologically invasive predator, the brown treesnake (Boiga irregularis). Zoo Biol. 2003;22(3):227–38. doi: 10.1002/zoo.10084

[50] Levine BA, Yackel Adams AA, Douglas MR, Douglas ME, Nafus MG. Female persistence during toxicant treatment predicts survival probability of offspring in invasive brown treesnakes (Boiga irregularis). Glob Ecol Conserv. 2021;31:e01827. doi: 10.1016/j.gecco.2021.e01827

[51] Nafus MG. Guam, USGS Closed Population (NWFN) data relating to brown treesnake and prey interactions processed into monthly intervals from 10/2016 - 2/2023. 2023.

[52] Lardner B, Savidge JA, Rodda GH. Spotting cryptic animals in the dark: What light properties should a good headlamp have? Managing Vertebrate Invasive Species. 2007;23:234–45.

[53] Rodda GH, Campbell EW. Distance sampling of forest snakes and lizards. Herpetol Rev. 2002;33(4):271–4.

[54] Caugley G. Analysis of vertebrate populations. Analysis of vertebrate populations. 1977:234.

[55] Siers SR, Nafus MG, Calaor JE, Volsteadt RM, Grassi MS, Volsteadt M, et al. Limitations of invasive snake control tools in the context of a new invasion on an island with abundant prey. NB. 2024;90 1–33. doi: 10.3897/neobiota.90.103041

[56] Gragg JE, Rodda GH, Savidge JA, White GC, Dean‐Bradley K, Ellingson AR. Response of brown treesnakes to reduction of their rodent prey. J Wildl Manag. 2007;71(7):2311–7. doi: 10.2193/2006-444

[57] Anderson DR. Response to Engeman: index values rarely constitute reliable information. Wildl Soc Bull. 2003;31(1):288–91.

[58] Anderson DR. The need to get the basics right in wildlife field studies. Wildl Soc Bull. 2001;29 1294–7.

[59] Boback SM, Nafus MG, Yackel Adams AA, Reed RN. Invasive brown treesnakes (Boiga irregularis) move short distances and have small activity areas in a high prey environment. Sci Rep. 2022;12(1):12705. doi: 10.1038/s41598-022-16660-y 35882893 PMC9325984

[60] Siers SR, Yackel Adams AA, Reed RN. Behavioral differences following ingestion of large meals and consequences for management of a harmful invasive snake: a field experiment. Ecol Evol. 2018;8(20):10075–93. doi: 10.1002/ece3.4480 30397449 PMC6206181

[61] Maunder MN, Sibert JR, Fonteneau A, Hampton J, Kleiber P, Harley SJ. Interpreting catch per unit effort data to assess the status of individual stocks and communities. ICES J Mar Sci 2006;63(8):1373–85. doi: 10.1016/j.icesjms.2006.05.008

[62] Johnston A, Hochachka WM, Strimas‐Mackey ME, Ruiz Gutierrez V, Robinson OJ, Miller ET, et al. Analytical guidelines to increase the value of community science data: an example using eBird data to estimate species distributions. Divers Distrib. 2021;27(7):1265–77. doi: 10.1111/ddi.13271

[63] Maunder MN, Thorson JT, Xu H, Oliveros-Ramos R, Hoyle SD, Tremblay-Boyer L, et al. The need for spatio-temporal modeling to determine catch-per-unit effort based indices of abundance and associated composition data for inclusion in stock assessment models. Fish Res. 2020;229:105594. doi: 10.1016/j.fishres.2020.105594

[64] Bauwens D, Claus K. Do newborn adders suffer mass mortality or do they venture into a collective hide-and-seek game? Biol J Linn Soc. 2018;124(1):99–112. doi: 10.1093/biolinnean/bly023

